# Tethered Antigenic Suppression Shields the Hemagglutinin Head Domain and Refocuses the Antibody Response to the Stalk Domain

**DOI:** 10.3390/chemistry7010012

**Published:** 2025-01-21

**Authors:** Donguk Kim, Kathryn Loeffler, Yixin Hu, Ammar Arsiwala, Steven Frey, Shruthi Murali, Vivek Hariharan, Alberto Moreno, Ravi S. Kane

**Affiliations:** 1 School of Chemical & Biomolecular Engineering, Georgia Institute of Technology, Atlanta, GA 30332, USA;; 2 Emory Vaccine Center, Emory National Primate Research Center, Emory University, Atlanta, GA 30329, USA;; 3 Division of Infectious Diseases, Department of Medicine, Emory University, Atlanta, GA 30322, USA

**Keywords:** vaccine, antigenic suppression, immune refocusing

## Abstract

Influenza has been a global health concern for the past century. Current seasonal influenza vaccines primarily elicit an antibody response that targets the immunodominant head domain of the viral glycoprotein hemagglutinin (HA), which consistently mutates due to selective pressure. To circumvent this problem, we introduce a “tethered antigenic suppression” strategy to shield the HA head domain and refocus the immune response towards the conserved but immunosubdominant stalk domain of HA. Specifically, we tethered an antibody fragment (Fab) that recognizes the Sb antigenic site in the HA head domain to the HA protein with a linker. We immunized separate groups of female mice with the Fab-tethered HA or regular HA and characterized the elicited antibody response. We demonstrate that shielding the HA head domain with a tethered Fab suppresses the antibody titers towards all five key antigenic sites in the HA head domain while eliciting a robust anti-stalk antibody response. Our work highlights the potential of tethered antigenic suppression as a strategy to refocus the antibody response towards conserved epitopes on protein antigens.

## 1. Introduction

Influenza remains a major global health challenge, causing up to 650,000 respiratory deaths worldwide [1]. Influenza A viruses, in particular, are pathogens that circulate between various hosts, including pigs, birds, horses, and humans [2], and have caused pandemics in the past, notably in 1918, but also in 1957, 1968, and more recently in 2009. Influenza A viruses are divided into subtypes according to the antigenic properties of hemagglutinin (HA) and neuraminidase (NA) viral surface glycoproteins. HA is the major antigenic protein of the influenza virus and is the primary target of the antibody response elicited by licensed seasonal influenza vaccines [3,4].

HA is composed of a highly variable head domain and a conserved stalk domain. The humoral immune response elicited by the current seasonal influenza vaccines primarily induces a narrow and strain-specific response that targets the globular head domain [5]. While the head-specific antibody response is often highly neutralizing and successfully inhibits viral attachment to host cells, the head domain undergoes rapid antigenic drift that enables viral escape from vaccine-induced immunity. Therefore, the strain-specific seasonal vaccines require annual reformulation [6]. Moreover, seasonal vaccines are unlikely to provide protection against novel influenza viruses caused by antigenic shift, a sudden and major change in antigenicity from the recombination of multiple human and non-human strains, which has led to influenza pandemics in the past [7]. It is thus imperative to develop a vaccine platform that provides robust and durable protection against a broad range of influenza strains and subtypes.

**Figure 1.**
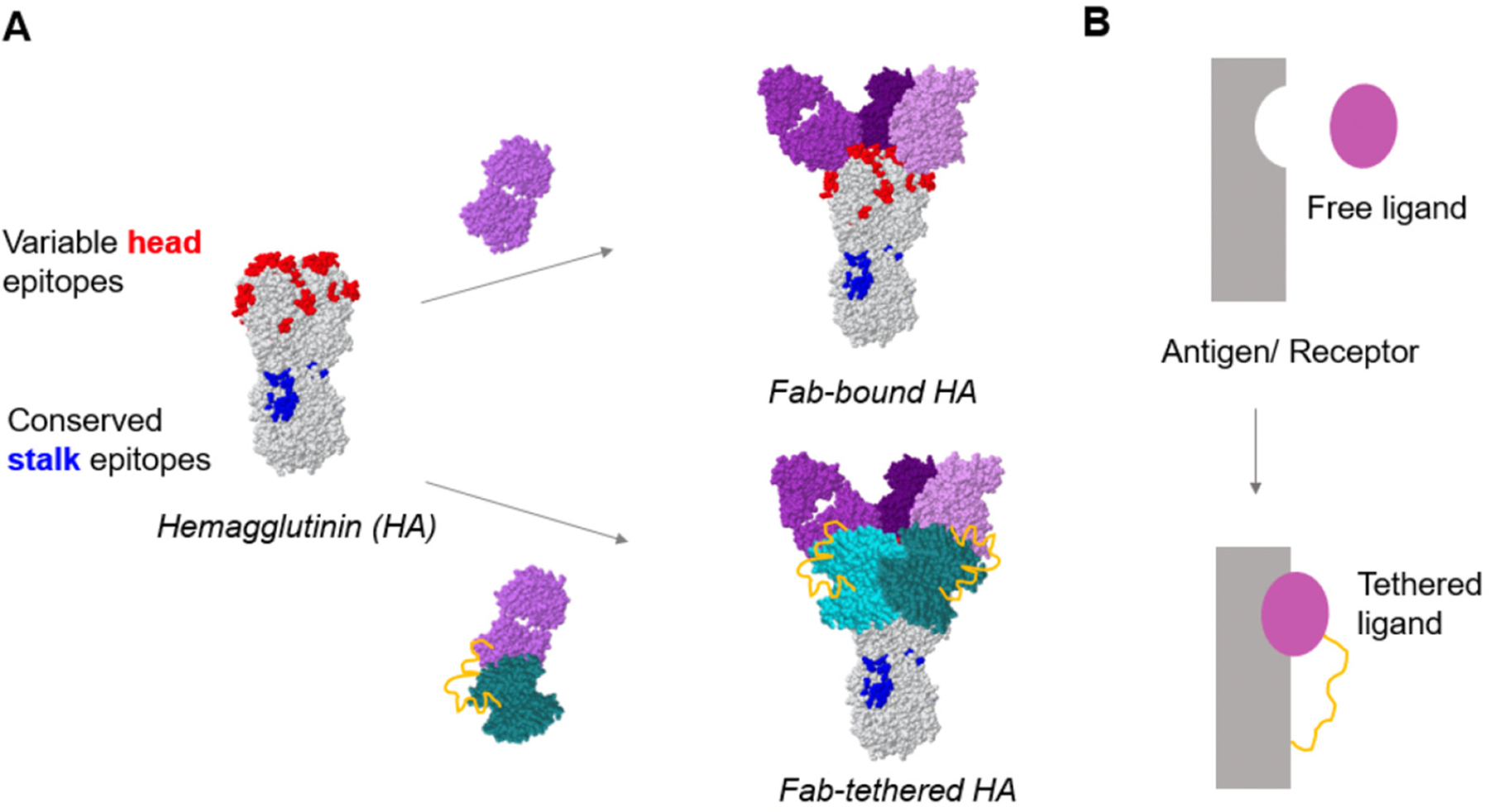
Schematic for tethering a Fab to HA to shield the head domain. (**A**) Top: HA bound to a H28-D14 Fab (purple) that binds to the Sb antigenic site located at the apex of the head region; bottom: H28-D14 Fab with a 5× GGGGS linker (yellow) tethered to HA via streptavidin (cyan). (**B**) Top: simplified schematic showing a free ligand and receptor pair, purple and gray, respectively; bottom: simplified schematic showing a tethered ligand (purple) with a linker (yellow) binding to a receptor (gray).

One strategy to achieve broader protection that has been the focus of numerous recent studies involves eliciting an immune response that targets the highly conserved stalk domain of HA [8–14]. The conceptually simplest and most elegant approach to generate a stalk-directed response would be to generate a stalk-only HA antigen [9,11,14]. While this approach holds considerable promise, generating a stable and correctly folded protein fragment is technically challenging and the approach is not readily generalizable to other protein antigens. In contrast, it is less challenging to express and stabilize the entire ectodomain of a protein antigen, such as HA, compared to a smaller fragment. When pursuing this second approach, a well-established strategy for refocusing the antibody response to a desired epitope on an antigen involves the introduction of “glycan shields” [15–17]. Along these lines, Eggink et al. hyperglycosylated the head domain of HA by introducing seven N-linked glycosylation sites to shield the immunodominant antigenic sites of the head domain. Doing so enhanced the stalk-directed response while reducing the head-directed response in immunized mice [8]. In this study, we present an alternative strategy for shielding the HA head domain using a tethered antibody fragment (Figure 1).

Our “tethered antigenic suppression” strategy is motivated by the concept of antigenic suppression, in which antibodies elicited by previous exposure to the antigen can mask their target epitope from memory immune cells. Angeletti et al. demonstrated that mixing inactivated A/PR/8/34 influenza virus with an antibody fragment (Fab) targeting the Sb antigenic site on the HA head suppressed the Sb-specific antibody response in immunized mice [18]. Chen et al. reported that forming a complex between the HIV-1 gp120 bridging sheet and an antibody targeting it reduces the immune response to this immunodominant region [19]. We hypothesized that Fabs could act as effective shields to selectively suppress antibody responses, not just against the Fab epitope, but also against other epitopes in the HA head (Figure 1A). Furthermore, from our prior analysis of multivalent ligand thermodynamics [20], we reasoned that the binding affinity of a Fab for its epitope could increase by tethering the Fab to the HA with a linker. This tethering mechanism would spatially restrict the Fab in the proximity of its antigen, thereby increasing the Fab’s effective concentration and its ability to bind and shield the antigenic site (Figure 1B). Here, we demonstrate that our tethered antigenic suppression approach effectively shields all five antigenic sites on the HA head and refocuses the antibody response towards the conserved stalk domain.

## 2. Materials and Methods

### 2.1. Expression and Purification of Avi-H28-D14 Fab

DNA encoding the variable regions of the heavy and light chains of H28-D14 (sequence provided by Dr. Jonathan Yewdell, National Institutes of Health) was synthesized and cloned into TGEX-FH and TGEX-LC vectors by Gene Universal, Inc. (Newark, DE, USA). The H28-D14 monoclonal antibody was generated by the immunization of a BALB/c mouse with the A/Puerto Rico/8/1934 influenza virus strain [21]. The TGEX-LC vector was modified to include an AviTag (GLNDIFEAQKIEWHE) followed by a 5× GGGGS linker at the N-terminus. The TGEX-FH vector was modified to include a hexa-histidine tag at the C-terminus. The two plasmids were then transformed into NEB 5-alpha *E. coli* (New England Biolabs, Ipswich, MA, USA) according to the user protocol for transformation. The transformants were plated on LB agar plates containing ampicilin (100 μg/mL) and incubated at 37 °C overnight. A colony was grown in 100 mL of LB media with ampicillin (100 μg/mL) overnight at 37 °C, and the plasmids were harvested using the PureLink HiPure Plasmid Filter Maxiprep Kit (Invitrogen, Waltham, MA, USA) according to the manufacturer’s instructions. The amplified plasmids were then transfected into Expi293F^™^ cells (Gibco, Waltham, MA, USA) using the ExpiFectamine 293 Transfection Kit (Gibco, Waltham, MA, USA) with a 2:1 light chain to heavy chain ratio, and the culture was incubated at 37 °C for five days. The culture was subsequently centrifuged at 7000× *g* for 7 min, and the supernatant containing the secreted Avi-H28-D14 Fab was dialyzed into PBS. The dialyzed supernatant was purified by immobilized metal affinity chromatography (IMAC) using a gravity flow column containing 1 mL of HisPur Ni-NTA resin (Thermo Scientific, Waltham, MA, USA). The resin was equilibrated with 20 column volumes (CVs) of binding buffer (50 mM Tris, 150 mM NaCl, 20 mM imidazole, pH 8.0) and incubated with the dialyzed supernatant overnight at 4 °C. The incubated resin was separated from the supernatant with the gravity flow column, washed with 40 CVs of binding buffer, and eluted three times with 5 CVs of elution buffer (150 mM Tris, 150 mM NaCl, 400 mM imidazole, pH 8.0) each time. The eluate was concentrated with a 10 kDa MWCO filter (MilliporeSigma, Burlington, MA, USA), injected into a size exclusion chromatography column (Superdex 200 Increase 10/300 GL, Cytiva, Marlborough, MA, USA), and eluted in a biotinylation buffer (20 mm Tris, 20 mm NaCl, pH 8.0).

### 2.2. Expression and Purification of AviTagged HA

The DNA encoding hemagglutinin (A/Puerto Rico/8/1934 H1N1) was optimized for mammalian cell expression, synthesized, and cloned into pcDNA 3.1(−) vector by Gene Universal Inc. (Newark, DE, USA). An AviTag was introduced between residues 77 and 78 using site-directed mutagenesis, and a trimerization domain from T4 phage fibritin followed by a hexa-histidine tag was inserted at the C-terminus. This plasmid was transfected into Expi293F cells, and the HA was purified using the same procedure as for the Avi-H28-D14 Fab.

### 2.3. In Vitro Biotinylation of Avi-H28-D14 Fab and HA

Avi-H28-D14 Fab and HA were biotinylated in vitro using the BirA500 kit (Avidity LLC, Aurora, CO, USA). Both proteins were diluted to 45 μM with the biotinylation buffer, then BirA and Biomix B (a proprietary solution containing biotin, adenosine triphosphate, and magnesium acetate) from the kit were added to the solutions according to the user protocol. The mixtures were shaken vigorously at 37 °C for 2 h, incubated at 4 °C overnight, and shaken at 37 °C for another 2 h. The biotinylated HA (b-HA) and H28-D14 Fab (b-H28-D14) were then purified by SEC and quantified with a BCA assay (Thermo Scientific, Waltham, MA, USA).

### 2.4. Expression and Purification of Streptavidin (SA)

SA was expressed, refolded, and purified as previously described [22–24]. pET21-Streptavidin-Glutamate_Tag was a gift from Mark Howarth (Addgene plasmid # 46367; http://n2t.net/addgene:46367 (accessed on 14 January 2025); RRID:Addgene_46367). In brief, streptavidin was expressed in BL21 *E. coli* (New England Biolabs, Ipswich, MA, USA) in 2xYT media as inclusion bodies before washing, unfolding in guanidine hydrochloride, and refolding via dropwise dilution in PBS. Streptavidin was then purified by ammonium sulfate precipitation and iminobiotin affinity chromatography, and concentration was determined using absorbance at 280 nm.

### 2.5. Assembly and Purification of SA-Fab from SA and b-H28-D14

SA-Fab was assembled by adding b-H28-D14 dropwise to a solution containing excess SA (4× molar ratio of SA to Fab) in a vial containing a stir bar while mixing at 1000 rpm. The assembled SA-Fab was loaded onto a HiLoad Superdex 200 PG preparative SEC column (Cytiva, Marlborough, MA, USA) to separate SA-Fab from excess SA. The SA-Fab fractions were collected, dialyzed into 20 mM Tris buffer (pH 8), and concentrated to less than 1 mL using a 10 kDa MWCO spin filter. To remove any unbiotinylated Avi-H28-D14, the concentrated SA-Fab was injected onto a MonoQ anion exchange column (Cytiva, Marlborough, MA, USA) and subjected to gradient elution with buffers containing 20 mM Tris (pH 8) and 20 mM Tris with 1 M NaCl (pH 8). The purified SA-Fab was dialyzed into PBS, concentrated, and quantified using a BCA assay.

### 2.6. Assembly of Fab-Tethered HA from SA-Fab and b-HA

To determine the optimal molar ratio of b-HA and SA-Fab that provides maximum coverage of HA without excess SA-Fab, b-HA was held constant at 1 μg, while SA-Fab was added at molar ratios ranging from 0.5 to 1.5. The proteins were mixed and allowed to incubate at room temperature for 20 min. The resulting mixtures were analyzed by SDS-PAGE by running at 120 V for 50 min. The optimal molar ratio was identified by selecting the ratio with minimal unconjugated HA. The Fab-tethered HA was subsequently assembled according to the determined ratio and was subjected to further characterization by SDS-PAGE, DLS, and SEC.

### 2.7. Dynamic Light Scattering (DLS)

DLS characterization was performed using a Zetasizer Nano (Malvern Panalytical, Malvern, Worcestshire, UK). Proteins were diluted up to 100 μL with PBS and pipetted into a UVette (Eppendorf, Hamburg, Germany and 10 acquisitions were collected per sample. Results were displayed as % volume versus diameter using the isotropic spheres model.

### 2.8. Expression and Purification of Regular HA

DNA encoding HA (A/Puerto Rico/8/1934 H1N1), codon optimized for mammalian cell expression, was synthesized and cloned into pcDNA 3.1(−) vector by Gene Universal Inc. (Newark, DE, USA). A myc tag (EQKLISEEDL) followed by a trimerization domain from T4 phage fibritin and a hexa-histidine tag were inserted at the C-terminus. This plasmid was cloned, transfected into Expi293F cells, and purified by IMAC using the same procedure as for the Avi-H28-D14 Fab. The HA was further purified by SEC using a Superdex 200 Increase 10/300 GL column (Cytiva, Marlborough, MA, USA) in PBS. The pure protein was quantified with a BCA assay.

### 2.9. In Vivo Vaccination with Fab-Tethered HA and Regular HA

Mice are the most widely used animal model in influenza research and were selected for immunogenicity assessment [25]. Female BALB/c (H-2^d^) mice, 6 to 8 weeks of age, were purchased from Charles River (Wilmington, MA, USA) and were acclimated for two weeks before use. The mice were housed in micro-isolation cages under daily oversight by veterinarians and veterinary technicians, randomized by minimization, and the sample size was calculated using the G*power 3.0.10 software. To test immunogenicity, mice were immunized with the Fab-tethered HA or HA control on day 0. For immunization, the proteins at 5 μg/ dose were formulated in the squalene-based oil-in-water adjuvant AddaVax (InvivoGen, San Diego, CA, USA) to a final volume of 100 μL, and the formulations were delivered subcutaneously in the base of the tail and the inter-scapular area. Blood samples were collected from tail snips 20 days after immunization. All procedures were performed by an investigator blinded to the experimental condition. During the follow-up, unusual signs were monitored clinically for evidence of distress. No exclusion criteria were set, and no animals were excluded. This study did not require the use of analgesics. All animal protocols were approved by Emory University’s Institutional Animal Care and Use Committee and followed institutional guidelines. The present study followed international, national, and institutional guidelines for humane animal treatment and complied with legislation from the Animal Welfare Act (AWA) and the American Association for Laboratory Animal Science guidelines.

### 2.10. Expression and Purification of Δ4 HAs

DNAs encoding Δ4 HAs [18], codon optimized for insect cell expression, were synthesized and cloned into pFastBacDual vector by GeneUniversal Inc. (Newark, DE, USA). A trimerization domain from T4 phage fibritin, followed by a hexa-histidine tag, was also included at the C-terminus. HA plasmids were transformed into DH10Bac competent cells (Gibco, Waltham, MA, USA), purified with E.Z.N.A.^®^ Plasmid DNA Mini Kit I, Q–spin, and transfected in Sf9 cells in Sf-900^™^ II SFM (Gibco, Waltham, MA, USA) using Cellfectin II (Gibco, Waltham, MA, USA). The P1 stock of the baculovirus was amplified to a P3 stock as described by Margine et al. [3]. Protein expression was performed in High Five^™^ Cells in Express Five Serum Free Medium (Gibco, supplemented with 18 mM L-glutamine). The culture was incubated at 28 °C for 4 days, and the supernatant containing the secreted Δ4 HAs was separated from the High Five cells through centrifugation (7000× *g*, 7 min). The supernatant was purified by IMAC using the same procedure as for the Avi-H28-D14 Fab and further purified by SEC using a Superdex 200 Increase 10/300 GL column (Cytiva, Marlborough, MA, USA) in PBS buffer. The purified proteins were quantified with a BCA assay.

### 2.11. Expression and Purification of Stalk-Only HA for Immunodepletion and ELISA

DNA encoding stalk-only HA (Gen 6 HA-SS, [14]), codon optimized for mammalian cell expression, was synthesized and cloned into pcDNA 3.1(−) vector by Gene Universal Inc. (Newark, DE, USA). A trimerization domain from T4 phage fibritin and a hexa-histidine tag was inserted at the C-terminus. This plasmid was cloned, transfected into Expi293F^™^ cells, and purified with IMAC using the same procedure as for the Avi-H28-D14 Fab. The stalk-only HA was further purified by SEC using a Superdex 200 Increase 10/300 GL column (Cytiva, Marlborough, MA, USA). The purified protein was quantified with a BCA assay.

### 2.12. Immunodepletions

For depletions with stalk-only HA, 500 μL of Ni-NTA beads were washed with IMAC Binding Buffer (50 mM Tris Base, 150 mM NaCl, 20 mM imidazole, pH 8.0) containing 1% BSA, incubated with the protein at 100 μg/mL, and allowed to bind to resin for two hours at 4 °C. The resin was washed with IMAC Binding Buffer-1% BSA and resuspended in 1.5 mL of the same buffer. Sera from individual mice were diluted 30,000-fold with 1% BSA in IMAC Binding Buffer. Sera samples were split into two tubes for incubation with resin containing the depletant protein or Ni-NTA beads without any protein immobilized. One hundred μL of resin slurry with or without depletant were added to the diluted sera and incubated for one hour at 4 °C on a 360° nutator. Incubation was followed by a 5 min centrifugation at 8000× *g*. The supernatants were transferred to a new tube, mixed with 100 μL of fresh resin slurry with or without depletant, and incubated for one hour at 4 °C on a 360° nutator. This process was repeated 3 times, with the last incubation conducted overnight at 4 °C on a 360° nutator. After the final immunodepletion, the depleted and undepleted sera were tested against full-length HA, stalk-only HA, and Δ4 HAs by ELISA.

### 2.13. Enzyme-Linked Immunosorbent Assays (ELISA)

The fine specificity of the antibodies elicited by immunization with the Fab-tethered HA and the regular HA control protein was determined by ELISA using Immulon 2HB plates (Thermo Labs Systems, Franklin, MA, USA) coated with 1 μg/mL of the recombinant proteins (regular HA, stalk-only HA, and Δ4 HAs) diluted in carbonate buffer (0.2 M sodium carbonate and 0.2 M sodium bicarbonate, pH: 9.6). The wells were blocked with a blocking buffer (1% bovine serum albumin (BSA) in PBS and 0.05% Tween 20) for two hours at 37 °C. Then duplicates of serum diluted in PBS with 0.5% BSA and 0.05% Tween 20 were added. The plates were then incubated for one hour at 37 °C, and the bound antibodies were then detected using peroxidase-labeled goat anti-mouse total IgG and SureBlue Reserve TMB as substrate (Sera Care, Milford, MA, USA). Optical densities (ODs) were determined using a VERSAmax ELISA reader (Molecular Device Corporation, Sunnyvale, CA, USA) with a 450 nm filter. Wells blocked with the blocking buffer without the addition of sera served as controls.

### 2.14. Avidity Assays

The relative avidity of antibodies was assessed by thiocyanate elution-based ELISA [26,27]. The assay was conducted similarly to the one described above, with slight modifications. In brief, 0 to 5M ammonium thiocyanate (NH_4_SCN) in PBS was added to each well after incubation with the sera dilutions. The plates were incubated for 15 min at room temperature before washing and proceeding with the assay described above. Control wells were incubated with the blocking buffer without NH_4_SCN. To determine the avidity index, the ODs that correspond to a 50% reduction of the ODs without NH_4_SCN were first defined. Then, four-parameter logistic regression was used to determine the molar concentration of NH_4_SCN that was responsible for the effect (concentration of the chaotropic agent eluting 50% of the bound antibodies).

## 3. Results and Discussion

### 3.1. Assembly of Fab-Tethered HA

The tethered antigenic suppression strategy (Figure 1) is based on shielding the HA head using a tethered Fab (H28-D14) targeting the Sb antigenic site on the HA head. In this study, we tethered the Fab to HA using streptavidin (SA). To enable this tethering, the light chain of the H28-D14 Fab was engineered to include a flexible 5× GGGGS peptide linker at its C-terminus followed by an AviTag, which allows for site-specific biotinylation. The biotinylated Fab was then allowed to bind to SA, creating a monovalent SA-Fab construct. In parallel, to create a biotinylation site on the HA head for SA binding, an AviTag was inserted between residues 77 and 78 of HA within the Cb antigenic site [28]. Following biotinylation, the biotinylated HA (b-HA) was assembled with the SA-Fab ligand to form Fab-tethered HA. We note that an AviTag insertion was used to facilitate tethering in this study because the N and C-termini of HA are distant from the antigenic sites in the head domain.

**Figure 2.**
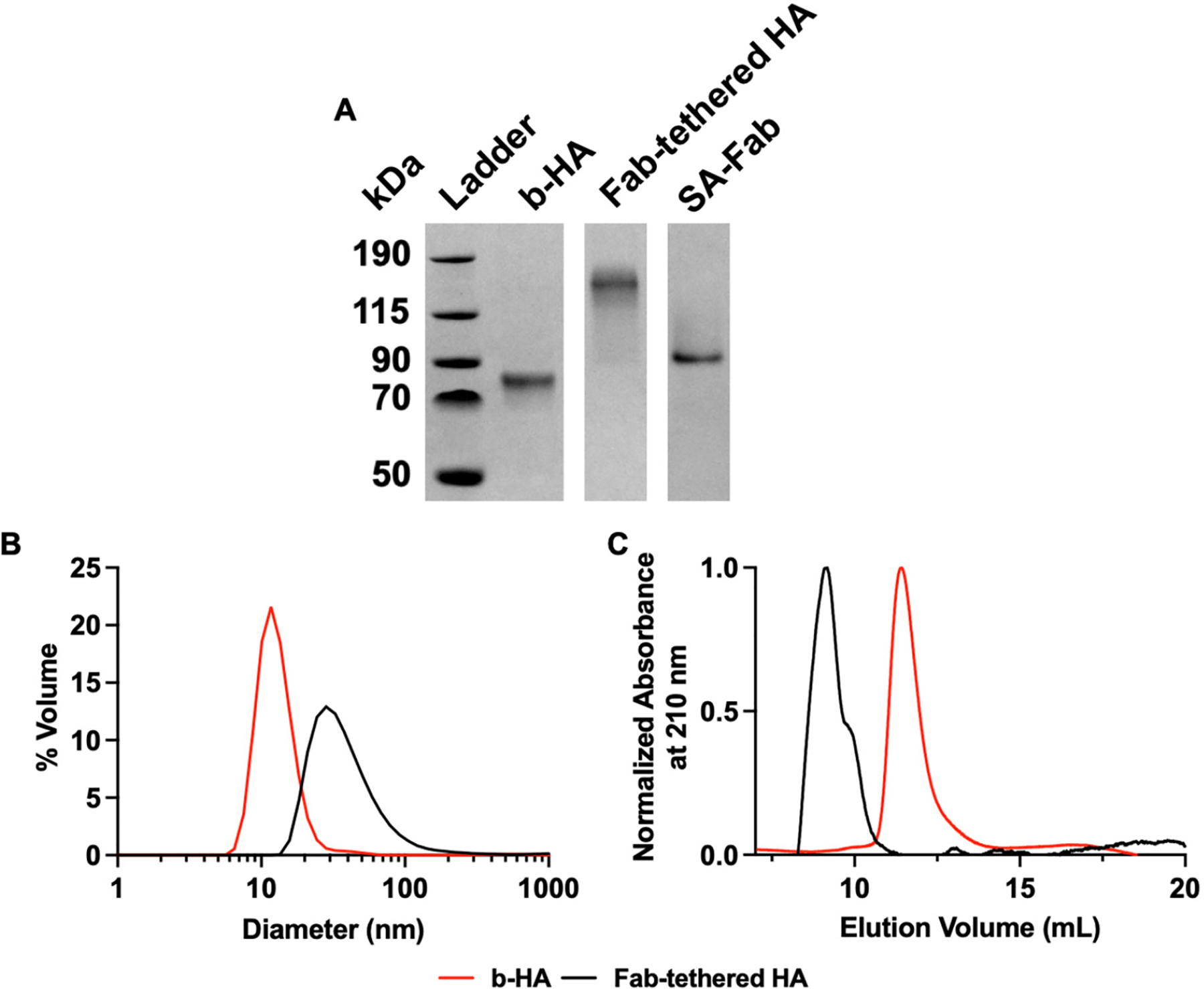
In vitro characterization of Fab-tethered HA assembly. (**A**) SDS-PAGE characterization of proteins used to assemble the Fab-tethered HA. The unprocessed image is provided in Supplementary Information Figure S1. (**B**) Dynamic light scattering (DLS) analysis of b-HA and Fab-tethered HA. (**C**) Size exclusion chromatography traces for b-HA and Fab-tethered HA.

We characterized the assembly of Fab-tethered HA using SDS-PAGE, dynamic light scattering (DLS), and size exclusion chromatography (SEC). As shown in Figure 2A, the Fab-tethered HA and its components migrated as expected based on their molecular weights. The expected molecular weight of monomeric HA is 70 kDa and Fab-tethered HA is about 170 kDa, which matches the result shown in the gel. Moreover, characterization by DLS (Figure 2B) indicated diameters of 13.2 nm for HA alone and 32.7 nm for Fab-tethered HA, which were consistent with expectations. The assembly is further supported by the SEC chromatogram (Figure 2C), as the Fab-tethered HA elutes earlier (~8.5 mL) compared to b-HA (~12 mL).

### 3.2. Immunization with Fab-Tethered HA and Characterization of Shielding of the HA Head Domain

With the confirmation of the assembly of Fab-tethered HA, we subsequently performed in vivo characterization of its immunogenicity. BALB/c mice were immunized with 5 μg of regular HA (A/Puerto Rico/8/1934) or Fab-tethered HA (equivalent to 5 μg of HA). Vaccine sample volumes were adjusted to 50 μL with PBS, and an equal volume of AddaVax adjuvant was mixed with each sample. Groups of five mice were vaccinated with the sample/AddaVax mixture, and sera were collected 20 days post-immunization.

**Figure 3.**
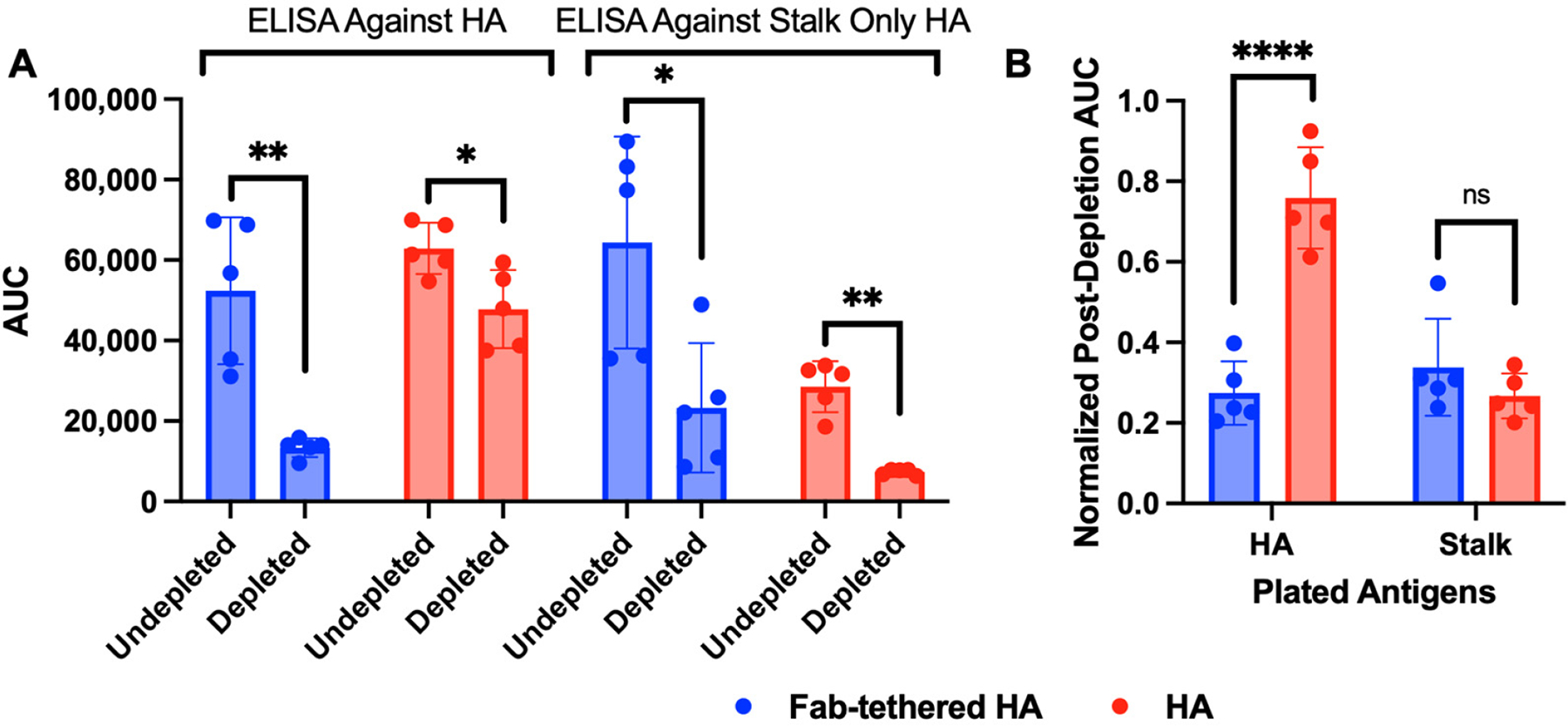
Characterization of sera of BALB/c mice vaccinated with Fab-tethered HA and HA. (**A**) Area under the ELISA curve of vaccinated sera pre-depletion and post-depletion with stalk-only HA tested against full-length HA and stalk-only HA. Bar graphs represent geometric mean with geometric standard deviation; *n* = 5 mice immunized with Fab-tethered HA; *n* = 5 mice immunized with regular HA. ** *p* < 0.01 and * *p* < 0.05 were determined by Welch’s *t*-test (α = 0.05). (**B**) Normalized post-depletion AUC—the ratio of the AUC values post-depletion and pre-depletion—for the vaccinated mice sera against full-length HA and stalk-only HA. Bar graphs represent geometric mean with geometric standard deviation; *n* = 5 mice immunized with Fab-tethered HA; *n* = 5 mice immunized with regular HA. No significance (ns) and **** *p* < 0.0001 were determined by unpaired two-tailed *t*-test (α = 0.05).

To determine whether the tethered Fab shield successfully refocused the immune response away from the HA head, we performed immunodepletion of sera using stalk-only HA (Gen6-HA-SS, [14]) to remove the stalk-targeting antibodies. The area under the ELISA curve (AUC) of sera against full-length HA and stalk-only HA was calculated to measure the level of the anti-HA antibodies pre- and post-immunodepletion (Figure 3A). Additionally, to account for differences in initial antibody titers in the undepleted sera, the AUC of the depleted sera was also plotted after normalization against the AUC of the undepleted sera (normalized post-depletion AUC; Figure 3B). Since the depleted sera should retain only antibodies targeting the HA head, a lower normalized post-depletion AUC indicates a lower fraction of head-targeting antibodies in the undepleted sera. As shown in Figure 3B, sera from mice vaccinated with Fab-tethered HA showed a much lower normalized post-depletion AUC against HA compared to sera from HA-vaccinated mice, indicating that the antibodies elicited by Fab-tethered HA primarily targeted the HA stalk.

**Figure 4.**
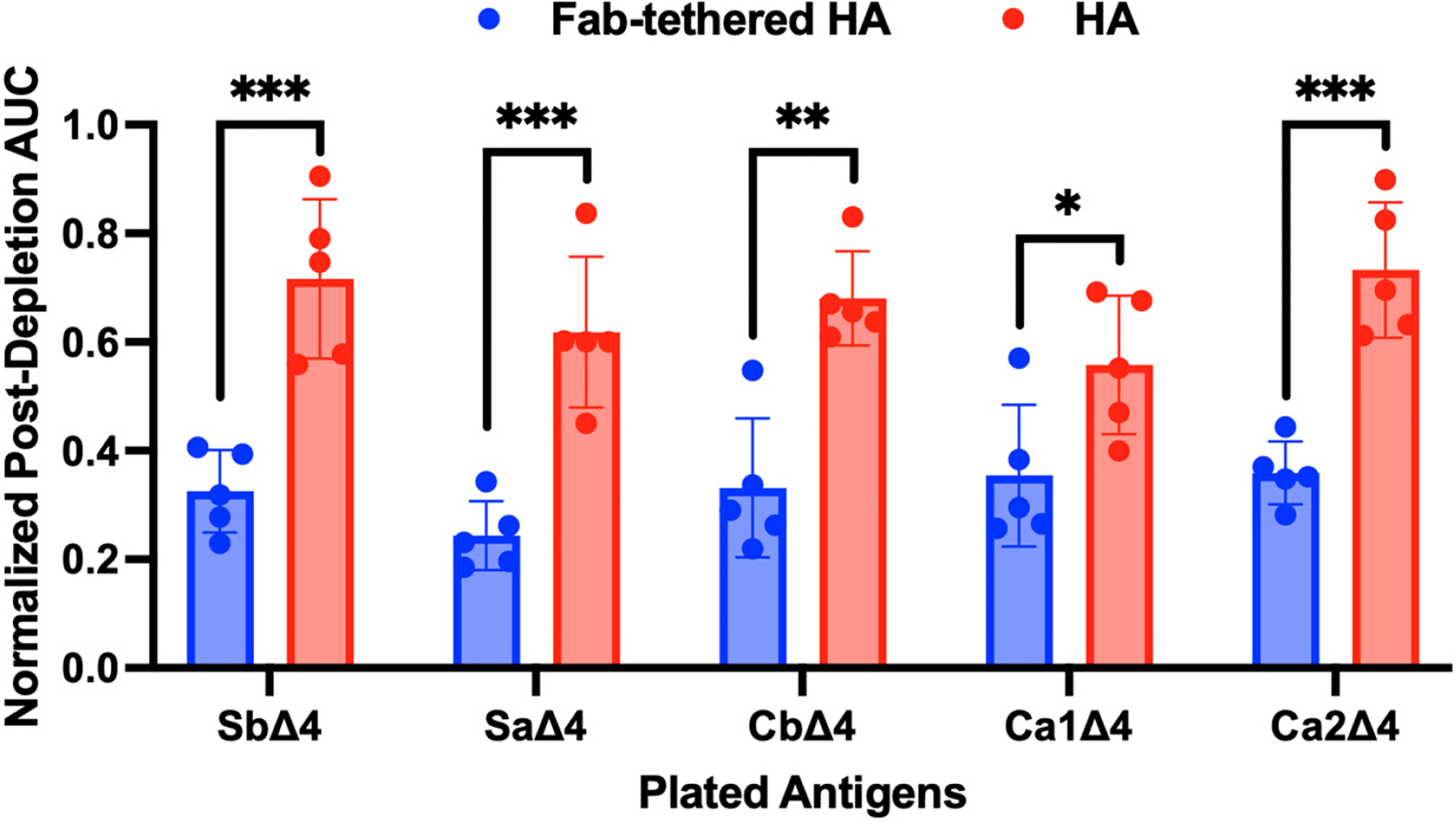
Characterization of shielding of key antigenic sites on the HA head domain by immunodepletion. Normalized post-depletion AUC for sera of mice immunized with Fab-tethered HA and HA against Δ4 HAs. The raw ELISA curve and AUC values are shown in Figures S3 and S4, respectively. Bar graphs represent geometric mean with geometric standard deviation; *n* = 5 mice immunized with Fab-tethered HA; *n* = 5 mice immunized with regular HA. *** *p* < 0.001, ** *p* < 0.01, and * *p* < 0.05 were determined by unpaired two-tailed *t*-test (α = 0.05).

Building on these promising results, we sought to further investigate the shielding profile of the Fab-tethered HA. Previous studies have identified five major antigenic sites on the HA head that serve as primary targets of neutralizing antibodies [28]: Sb and Sa, located on the top of the globular HA head, and Cb, Ca1, and Ca2, positioned on the lateral regions. Based on these antigenic sites, Angeletti et al. developed Δ4 HA constructs in which one antigenic site remains intact while the other four are mutated to reduce their immunogenicity [18]. Our tethered Fab design was expected to effectively shield the Sb and Cb sites through the binding of H28-D14 Fab and streptavidin (SA), respectively. However, it was unclear whether the remaining antigenic sites (Sa, Ca1, and Ca2) would also be shielded in the Fab-tethered HA. To evaluate the shielding of all antigenic sites, we performed ELISAs using Δ4 HA constructs and the same sera from mice vaccinated with either Fab-tethered HA or regular HA. Similar to our analysis with full-length HA and stalk-only HA, we analyzed the immunodepleted and undepleted sera for antibodies targeting the remaining antigenic site on each Δ4 HA. In this analysis, a lower normalized post-depletion AUC suggests a lower fraction of antibodies targeting the corresponding antigenic site in the undepleted sera. As shown in Figure 4, the normalized post-depletion AUCs for all antigenic sites in the Fab-tethered HA were significantly lower compared to the regular HA, indicating effective shielding of all five sites.

**Figure 5.**
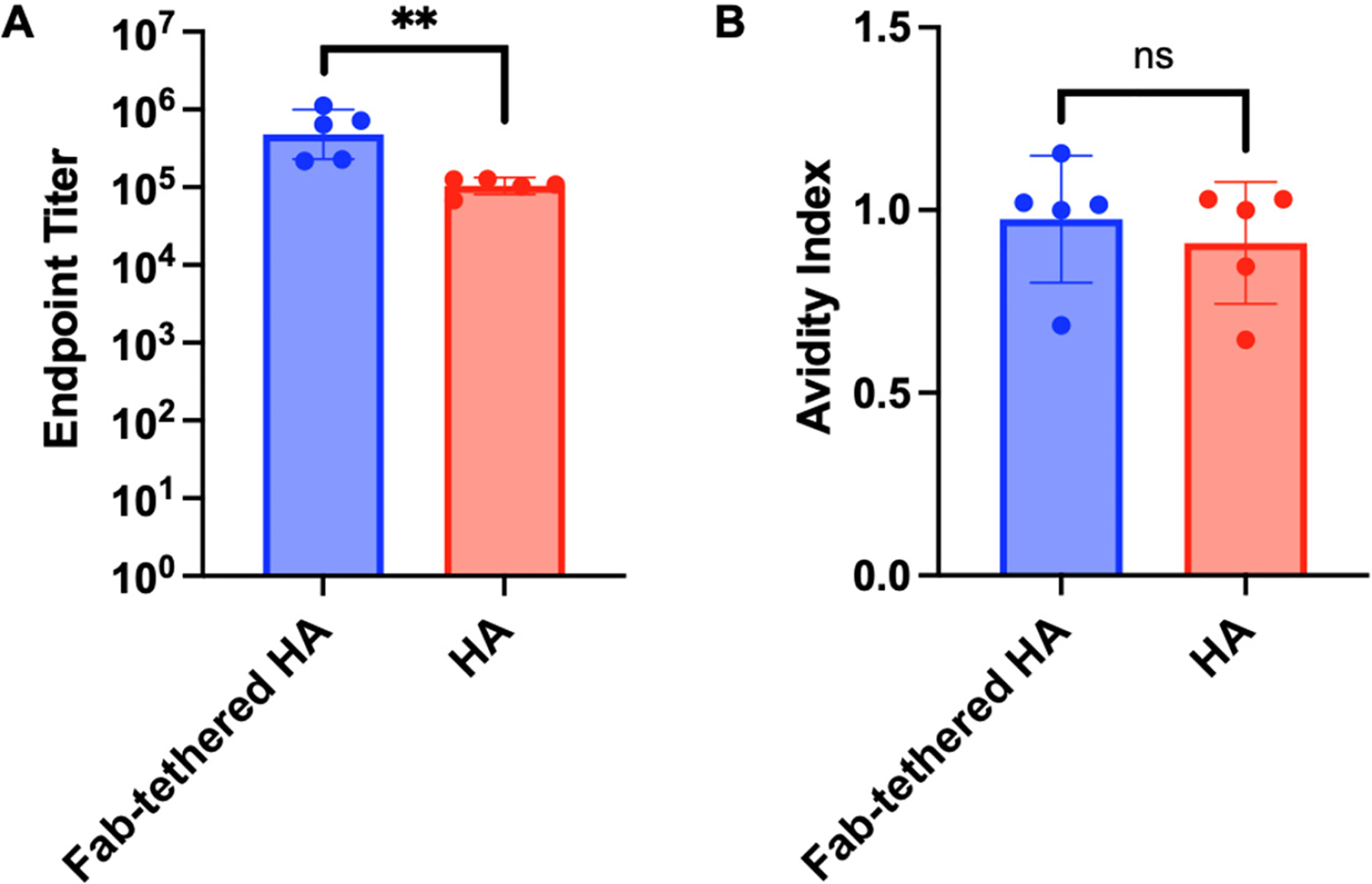
Characterization of the anti-stalk antibody response. (**A**) Endpoint antibody titers against stalk-only HA for sera from mice vaccinated with Fab-tethered HA and HA. The raw ELISA curve is shown in Figure S5. Antibody titers were determined by curve fitting with a four-parameter logistic regression in GraphPad Prism 10.4.1 using a cutoff of the mean absorbance plus three standard deviations of pre-immune samples. (**B**) Avidity index against stalk-only HA for sera from mice immunized with Fab-tethered HA and regular HA. Bar graphs represent geometric mean with geometric standard deviation; *n* = 5 mice immunized with Fab-tethered HA; *n* = 5 mice immunized with regular HA. ** *p* < 0.01 and no significance (ns) were determined by unpaired two-tailed *t*-test (α = 0.05).

Lastly, we evaluated whether immunizing mice with Fab-tethered HA resulted in a more robust immune response directed towards the HA stalk. ELISAs were performed using the same sera from mice vaccinated with Fab-tethered HA and regular HA against stalk-only HA. Sera from mice vaccinated with Fab-tethered HA exhibited significantly higher antibody titers against the HA stalk domain than sera from mice vaccinated with regular HA (Figure 5A). Moreover, the relative avidity of the stalk-targeting antibodies, assessed using a thiocyanate elution-based ELISA, remained unchanged (Figure 5B), indicating that the tethered Fab shielding strategy did not compromise the avidity of the stalk-targeting antibodies.

## 4. Conclusions

In this work, we have leveraged the concept of antigenic suppression to redirect the immune response away from the variable HA head domain and towards the conserved HA stalk domain. The results from the characterization of the Fab-tethered HA assembly and its immunogenicity demonstrate the effectiveness of the tethered antigenic suppression strategy. SDS-PAGE, DLS, and SEC confirmed the successful formation of the Fab-tethered HA complex, demonstrating the feasibility of the assembly method. Importantly, in vivo studies demonstrated that Fab-tethered HA successfully reduced head-targeted antibody responses and enhanced stalk-specific antibody production with no loss in anti-stalk antibody avidity. Additionally, Δ4 HA studies revealed the effective shielding of all five antigenic sites in the head domain. These findings highlight the potential of the tethered antigenic suppression strategy for designing broadly protective influenza vaccines, as H1 stalk-directed strategies have been shown to elicit broadly cross-reactive anti-HA antibodies [12,14,23].

## Supplementary Material

Supplementary Information

## Data Availability

The data that support the conclusions of this study are included in the article and supplementary material.
