# Supplementary material for: Tethered Antigenic Suppression Shields the Hemagglutinin Head Domain and Refocuses the Antibody Response to the Stalk Domain
Source: Chemistry (Basel). Author manuscript; Available in PMC 2025 Sep 27. (PMC12468577; doi:10.3390/chemistry7010012)
Supplement: Supplementary Information [file NIHMS2066010-supplement-Supplementary_Information.pdf]

Supporting Information

Tethered antigenic suppression shields the hemagglutinin head domain and refocuses the antibody response to the stalk domain

Donguk Kim[1], Kathryn Loeffler[1], Yixin Hu[1], Ammar Arsiwala[1], Steven Frey[1], Shruthi Murali[1], Vivek Hariharan[1], Alberto Moreno[2,3], and Ravi S. Kane[1,*]

[1] School of Chemical & Biomolecular Engineering, Georgia Institute of Technology, Atlanta, GA, USA.
[2] Emory Vaccine Center, Emory National Primate Research Center, Emory University, Atlanta, GA, USA.
[3] Division of Infectious Diseases, Department of Medicine, Emory University, Atlanta, GA, USA.
* Correspondence: ravi.kane@chbe.gatech.edu

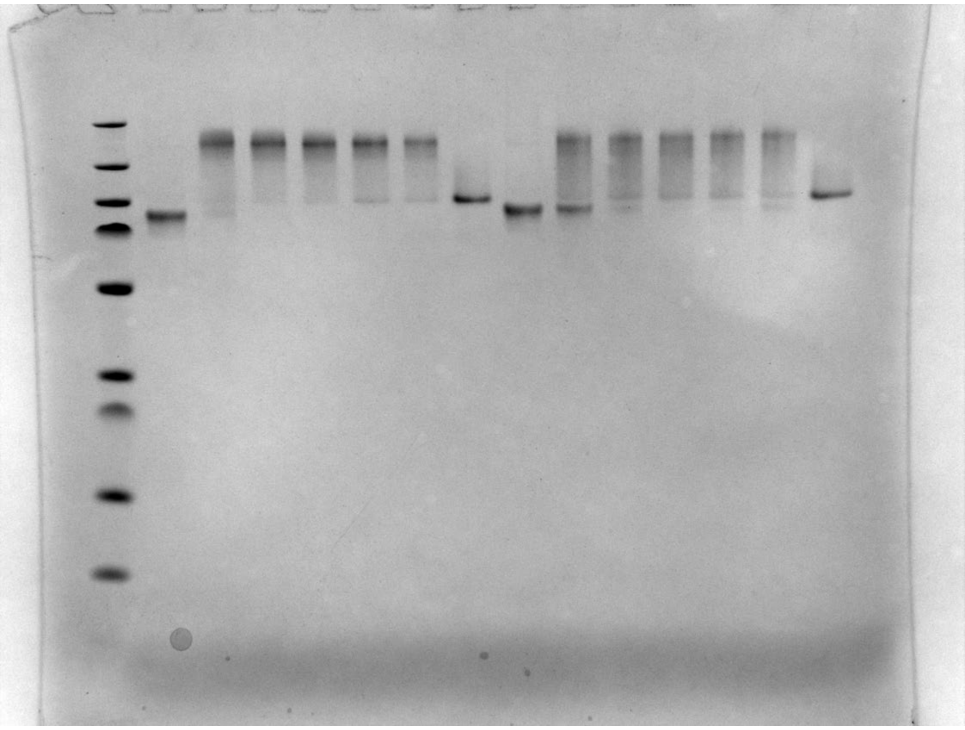

Figure S1. Unprocessed image of SDS-PAGE gel from Figure 2.

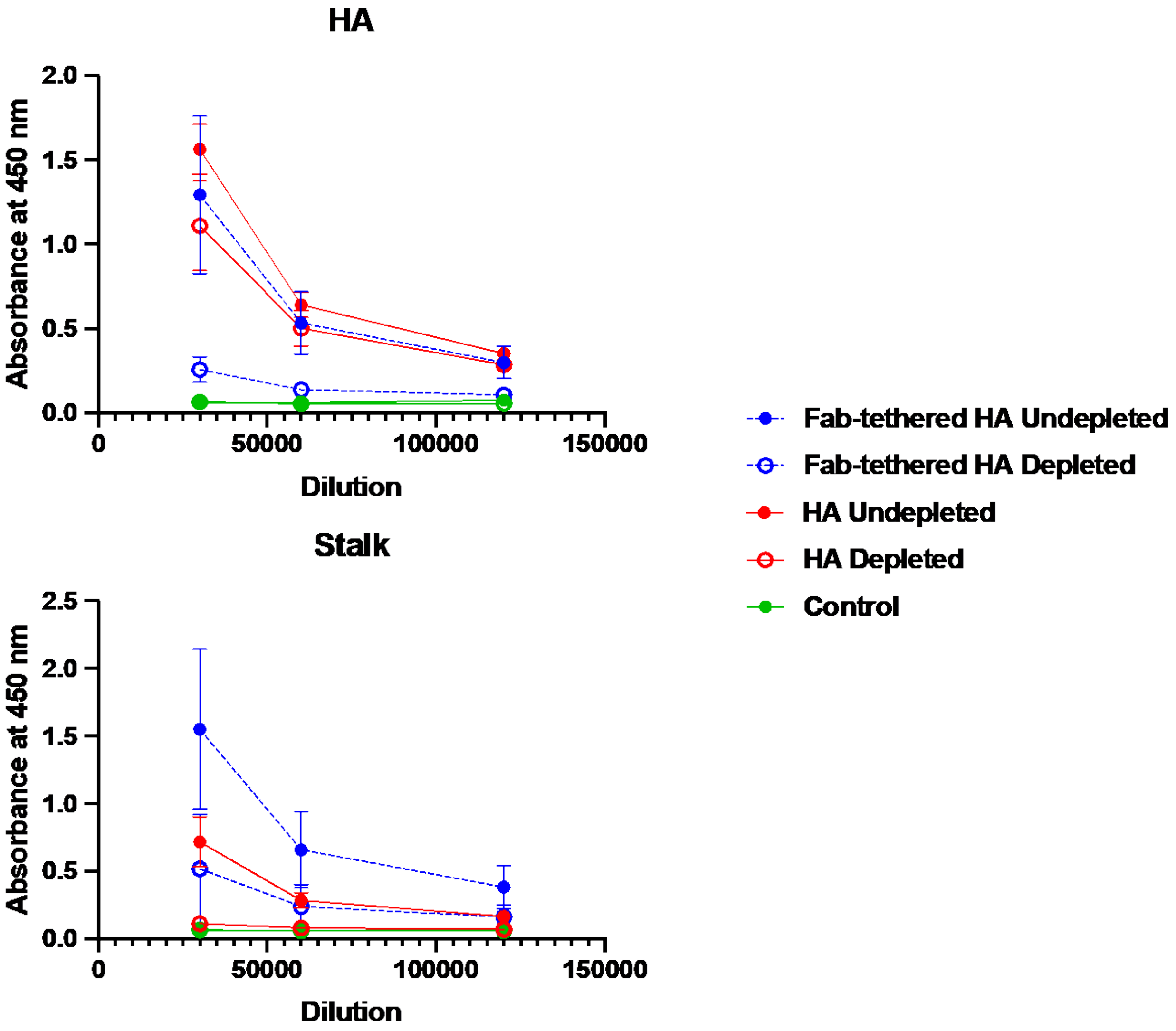

Figure S2. ELISA data for sera from vaccinated mice pre-depletion and post-depletion with stalk-only HA. The blocking buffer was used as a control (green) to determine the background values.

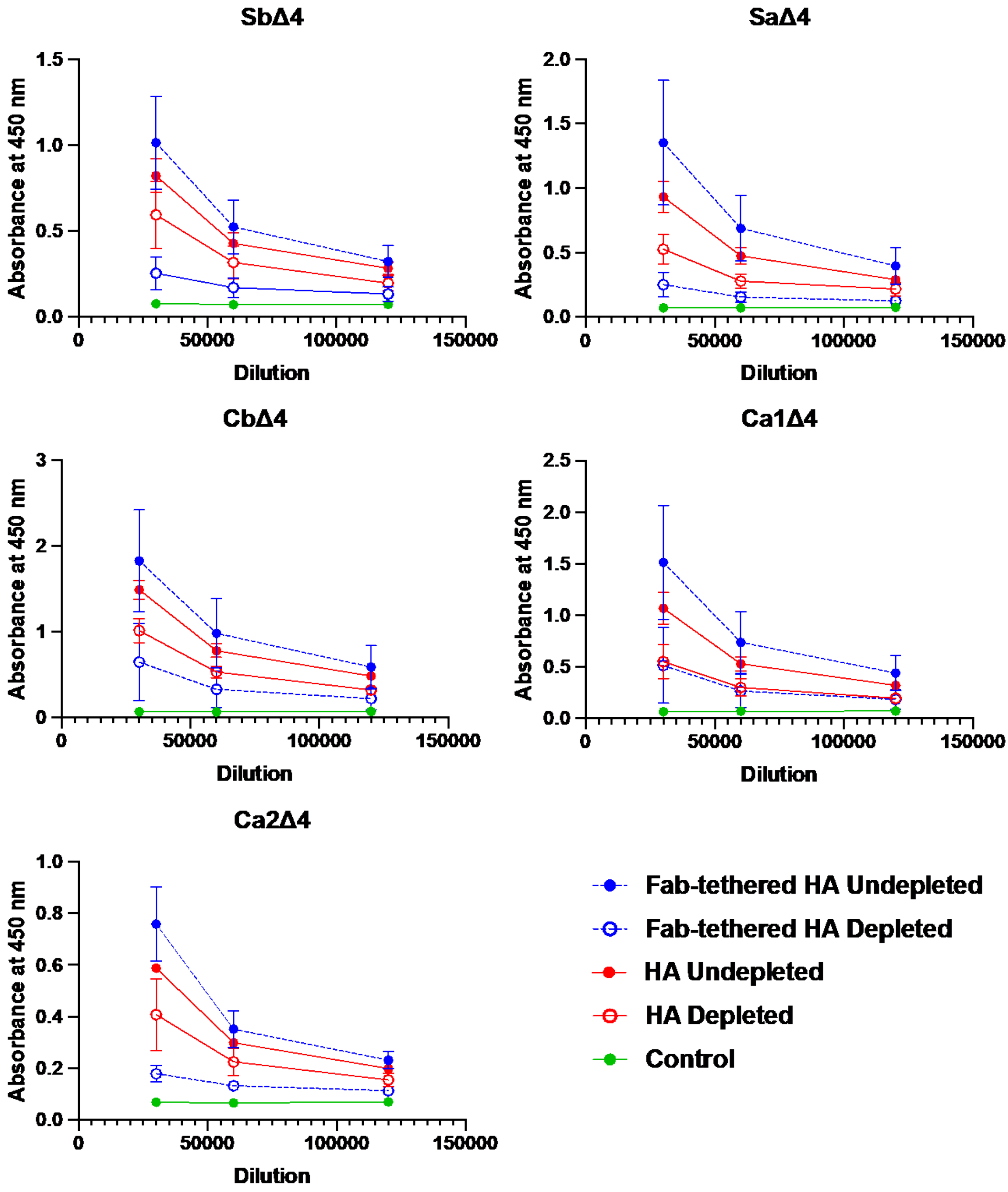

Figure S3. ELISA data against Δ4 HAs for sera from vaccinated mice pre-depletion and post-depletion with stalk-only HA. The blocking buffer was used as a control (green) to determine the background values.

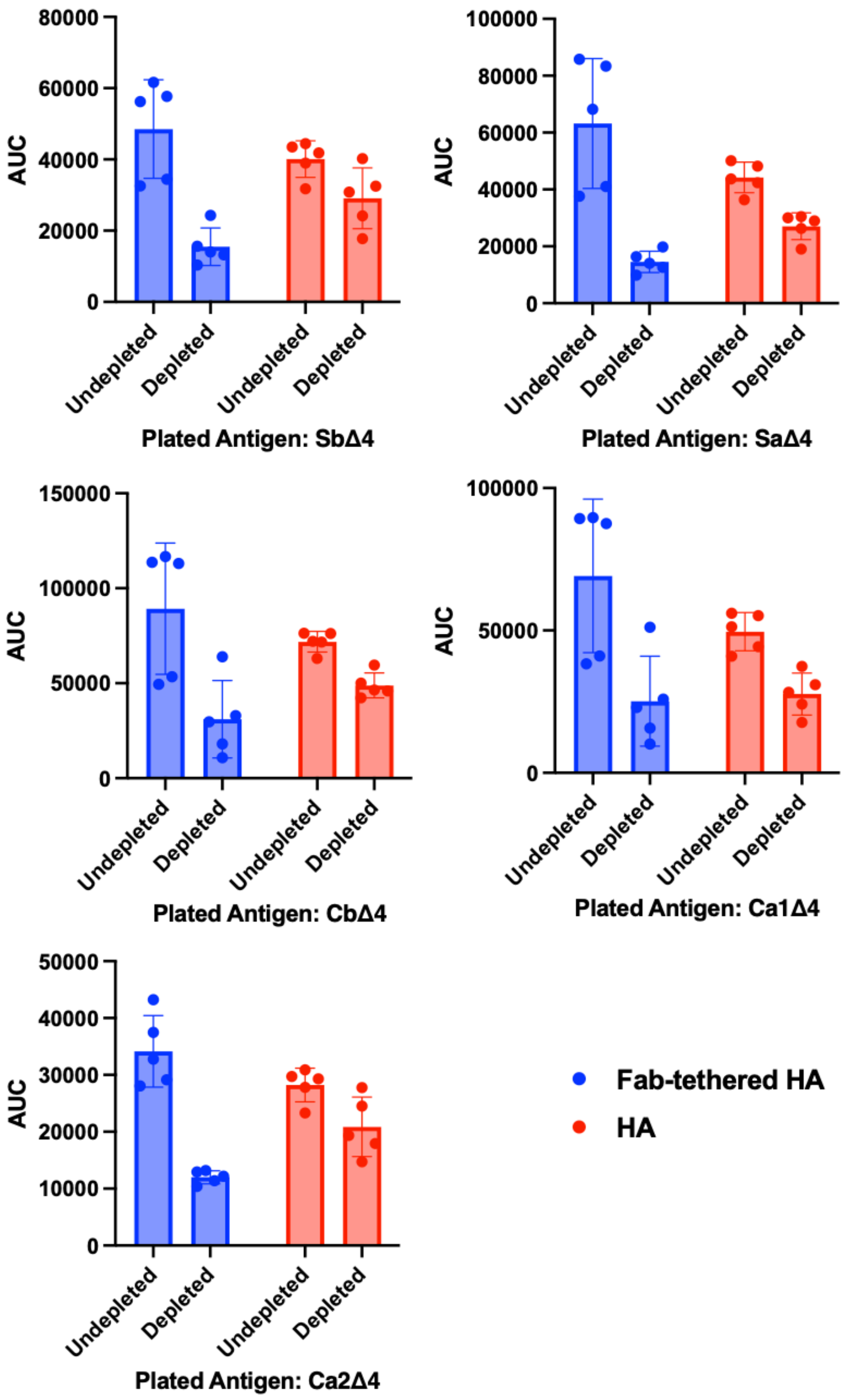

Figure S4. Area under the ELISA curve for sera from vaccinated mice pre-depletion and post-depletion with stalk-only HA tested against Δ4 HAs.

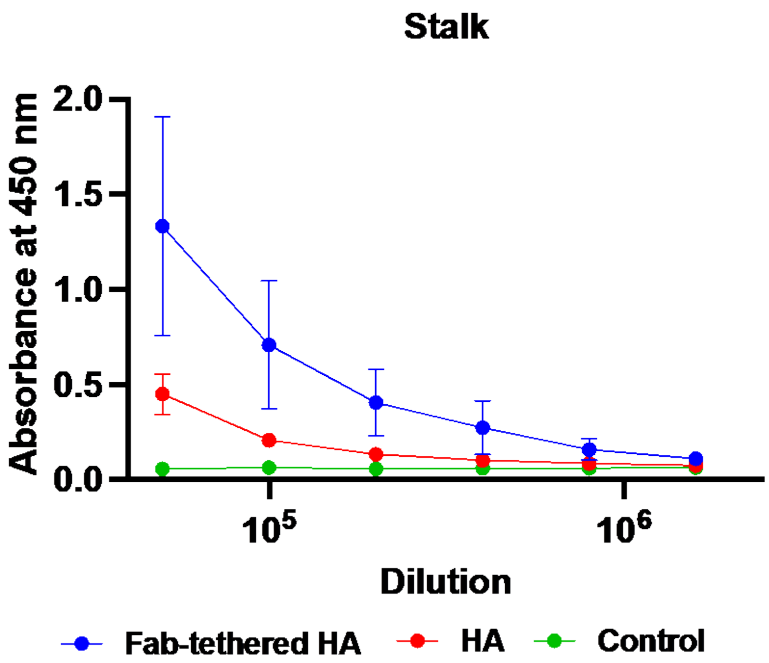

Figure S5. Measurement of anti-stalk titers by ELISA with sera from mice immunized with Fab-tethered HA (blue) and HA (red). The blocking buffer was used as a control (green).
